# Porphyria Cutanea Tarda: A Phenotypic Expression of Several Genes

**DOI:** 10.7759/cureus.83955

**Published:** 2025-05-12

**Authors:** Sebastián J Vázquez-Folch, Gabriel A Jimenez-Berrios, Natalio Izquierdo, Victor Vazquez

**Affiliations:** 1 School of Medicine, Universidad Central del Caribe, Bayamón, PRI; 2 Department of Surgery, School of Medicine, Medical Sciences Campus, University of Puerto Rico, San Juan, PRI; 3 Comprehensive Cancer Center, School of Medicine, Medical Sciences Campus, University of Puerto Rico, San Juan, PRI

**Keywords:** erythropoietic protoporphyria, erythropoietin, hemochromatosis, porphyria cutanea tarda, uroporphyrinogen decarboxylase

## Abstract

Porphyria comprises a group of rare inherited or acquired disorders characterized by defects in the heme biosynthetic pathway, resulting in the accumulation of porphyrins or their precursors. This study presents three cases of porphyria in Puerto Rico, including erythropoietic protoporphyria (EPP) and porphyria cutanea tarda (PCT). Genetic testing revealed a heterozygous mutation in the FECH gene in the EPP case and an HFE gene mutation in a PCT case with hereditary hemochromatosis. A previously undocumented case of PCT with elevated uroporphyrin levels but negative genetic panel results raises questions about the genetic basis of porphyria. Our findings highlight the importance of genetic testing in diagnosing and managing porphyria, emphasizing the need for further research into its genetic and phenotypic diversity. This study contributes to the understanding of porphyria in Puerto Rico, offering insights into its clinical and genetic complexities.

## Introduction

Porphyria is a group of rare inherited or acquired disorders resulting from the malfunction of the heme biosynthetic pathway, leading to the accumulation of porphyrins or their precursors [1,2]. The heme biosynthetic pathway is crucial for the production of heme, an essential component of hemoglobin, myoglobin, and various cytochromes [3]. Defects in this pathway may result in a variety of clinical manifestations, including the skin and nervous system [4].

Porphyrias are classified and include erythropoietic protoporphyria (EPP), porphyria cutanea tarda (PCT), acute intermittent porphyria, hereditary coproporphyria, variegate porphyria, and congenital erythropoietic porphyria [5].

EPP is primarily caused by autosomal recessive mutations in the FECH gene, which encodes the enzyme ferrochelatase [6]. This enzyme is responsible for the last step in heme production, where iron is inserted into protoporphyrin IX to form heme [7]. A deficiency in ferrochelatase leads to the accumulation of protoporphyrin IX in erythrocytes, plasma, and tissues. Patients with EPP typically have photosensitivity, experiencing burning pain and erythema upon exposure to sunlight. Upon chronicity, the disease may lead to liver complications due to the buildup of protoporphyrins in the bile [6].

PCT is the most common type of porphyria and is usually acquired, although it may also have a genetic inheritance. PCT is characterized by a deficiency in the enzyme uroporphyrinogen decarboxylase (UROD), which leads to the accumulation of uroporphyrins in the liver, plasma, and skin [8]. This accumulation causes photosensitivity, resulting in painful blistering, fragility, and hyperpigmentation of sun-exposed skin areas [9]. PCT can be associated with liver dysfunction and iron overload, and it is often linked with genetic variants such as mutations in the HFE gene, leading to hereditary hemochromatosis [10]. An increased frequency of hereditary hemochromatosis gene mutations occurs in patients with PCT [11].

## Case presentation

The key findings of the patients are summarized in Table 1.

**Table 1:** Clinical description, treatment, and therapeutic response in patients with porphyria F: female; M: male

Case number	Age/sex	Type of porphyria	Gene	Mutation	Organ involvement	Initial treatment	Response to initial treatment
1	68/F	Erythropoietic protoporphyria	FECH gene	(c.315-48T>C)	Skin	Phlebotomy	Responsive
2	41/M	Inconclusive	Inconclusive	Inconclusive	Skin	Phlebotomy	Developed intolerance; consider hydroxychloroquine
3	70/M	Porphyria cutanea tarda	HFE gene	C282Y (c.845G>A, p.Cys282Tyr) and H63D (C.187C>G, p.His63Asp)	Skin	Phlebotomy	Responsive

Case 1: EPP

A 68-year-old female patient had skin lesions in sunlight-exposed areas and pruritus urticaria and was diagnosed with porphyria in July 2010. She was referred by her dermatologist for therapeutic phlebotomy. Her laboratory results showed elevated erythropoietin levels at 43.65 mIU/mL. Upon genetic testing (Invitae, San Francisco, California, United States), the patient had a heterozygous mutation in the FECH gene (c.315-48T>C). FibroScan (Echosens, Paris, France) was completed in December 2023. The liver stiffness measurement (LSM) was 5.2 kPa. The patient was diagnosed with minimal hepatic fibrosis. Laboratory test results are shown in Tables 2-3.

**Table 2:** Case 1: lab results pre-phlebotomy (2018) μg: micrograms; L: liters; hr: hours

Laboratory test results pre-phlebotomy	
Uroporphyrins (UP)	5 ug/L
Heptacarboxyl (7-CP)	1 μg/L
Hexacarboxyl (6-CP)	43 μg/L
Hexacarboxyl (6-CP), 24 hr	75 μg/24 hr (HIGH)
Pentacarboxyl (5-CP)	<1 μg/L
Coproporphyrin (CP) I	8 μg/L
Coproporphyrin (CP) III	33 μg/L

**Table 3:** Case 1: lab results post-phlebotomy (2024) μg: micrograms; L: liters; hr: hours

Laboratory test results post-phlebotomy	
Uroporphyrins (UP)	6 μg/L
Heptacarboxyl (7-CP)	<1 μg/L
Hexacarboxyl (6-CP)	<1 μg/L
Pentacarboxyl (5-CP)	4 μg/L
Pentacarboxyl (5-CP), 24 hr	10 μg/24 hr (HIGH)
Coproporphyrin (CP) I	6 μg/L
Coproporphyrin (CP) III	13 μg/L

**Figure 1:**
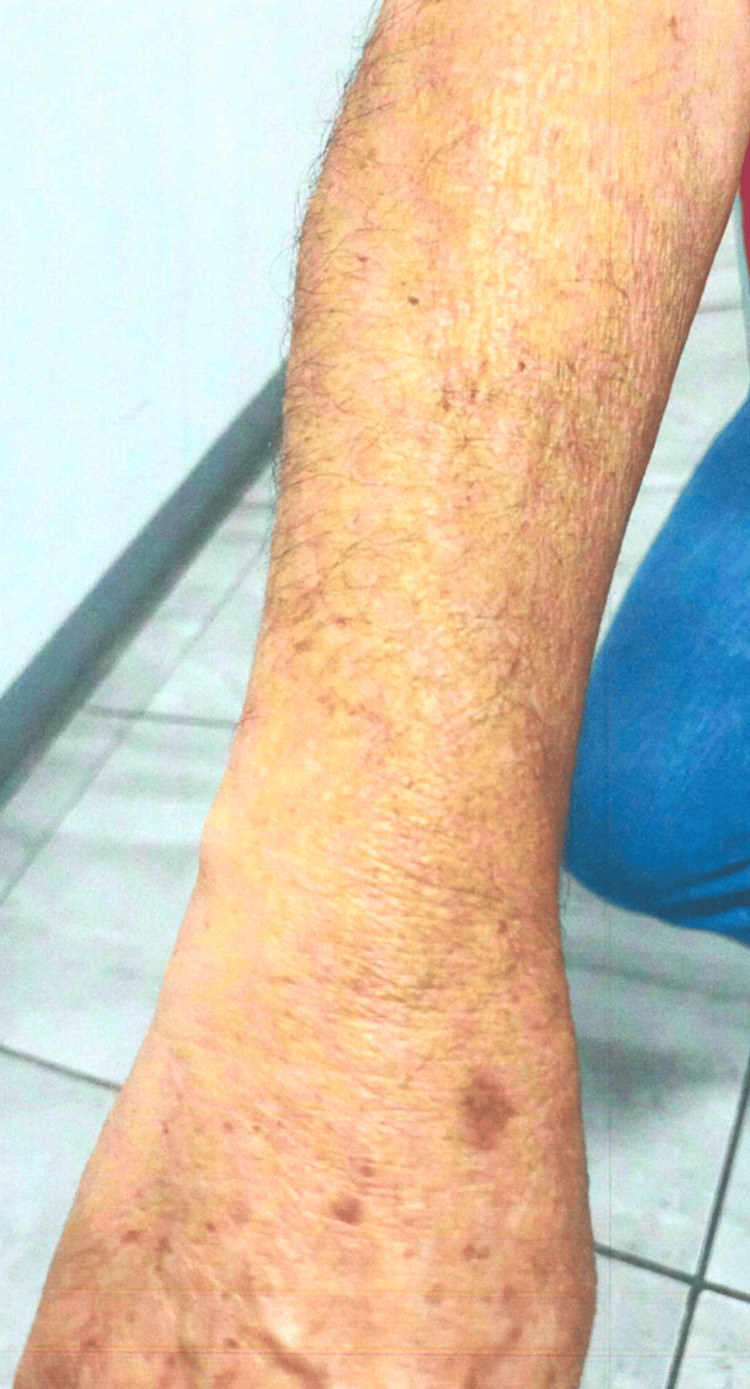
Case 1: patient's dermatologic findings in the right arm

**Figure 2:**
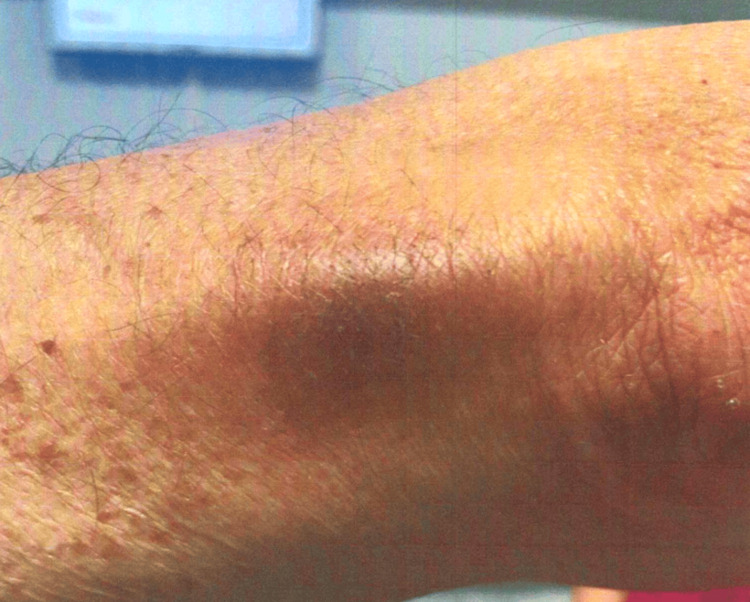
Case 1: patient's dermatologic findings in the left upper extremity

The patient's dermatologic findings are shown in Figures 1-2.

Case 2: PCT

Case 1's offspring, a 41-year-old male patient, had a history of skin blistering and photosensitivity. He was diagnosed with PCT in 2010 and was referred by his dermatologist for phlebotomy, which lowered his hemoglobin to 10 g/dL. His symptoms improved post-phlebotomy. His laboratory results in 2010 showed elevated levels of various porphyrins, including uroporphyrins and coproporphyrins. His erythropoietin levels are high, 19.37 mIU/mL, in 2024. Genetic studies (Invitae) showed no mutations in the porphyria panel. The patient tested negative for the following porphyria enzymes: ALAD, ALAS2, CPLX, CPOX, FECH, GATA1, HMBS, PPOX, UROD, and UROS. He developed intolerance to phlebotomy due to dizziness, tachycardia, and hypotension. Therefore, the patient was advised to follow up with quantitative porphyrins and consider starting low doses of hydroxychloroquine (Plaquenil) for symptom management. Laboratory test results are shown in Tables 4-5.

**Table 4:** Case 2: lab results pre-phlebotomy (2010) μg: micrograms; L: liters

Laboratory test results pre-phlebotomy	
Uroporphyrins (UP)	21 μg/L (HIGH)
Heptacarboxyl (7-CP)	1 μg/L
Hexacarboxyl (6-CP)	1 μg/L
Pentacarboxyl (5-CP)	2 μg/L
Coproporphyrin (CP) I	11 μg/L
Coproporphyrin (CP) III	6 μg/L

**Table 5:** Case 2: lab results post-phlebotomy (2024) μg: micrograms; L: liters

Laboratory test results post-phlebotomy	
Uroporphyrins (UP)	11 μg/L
Heptacarboxyl (7-CP)	<1 μg/L
Hexacarboxyl (6-CP)	<1 μg/L
Pentacarboxyl (5-CP)	<1 μg/L
Coproporphyrin (CP) I	13 μg/L
Coproporphyrin (CP) III	29 μg/L

**Figure 3:**
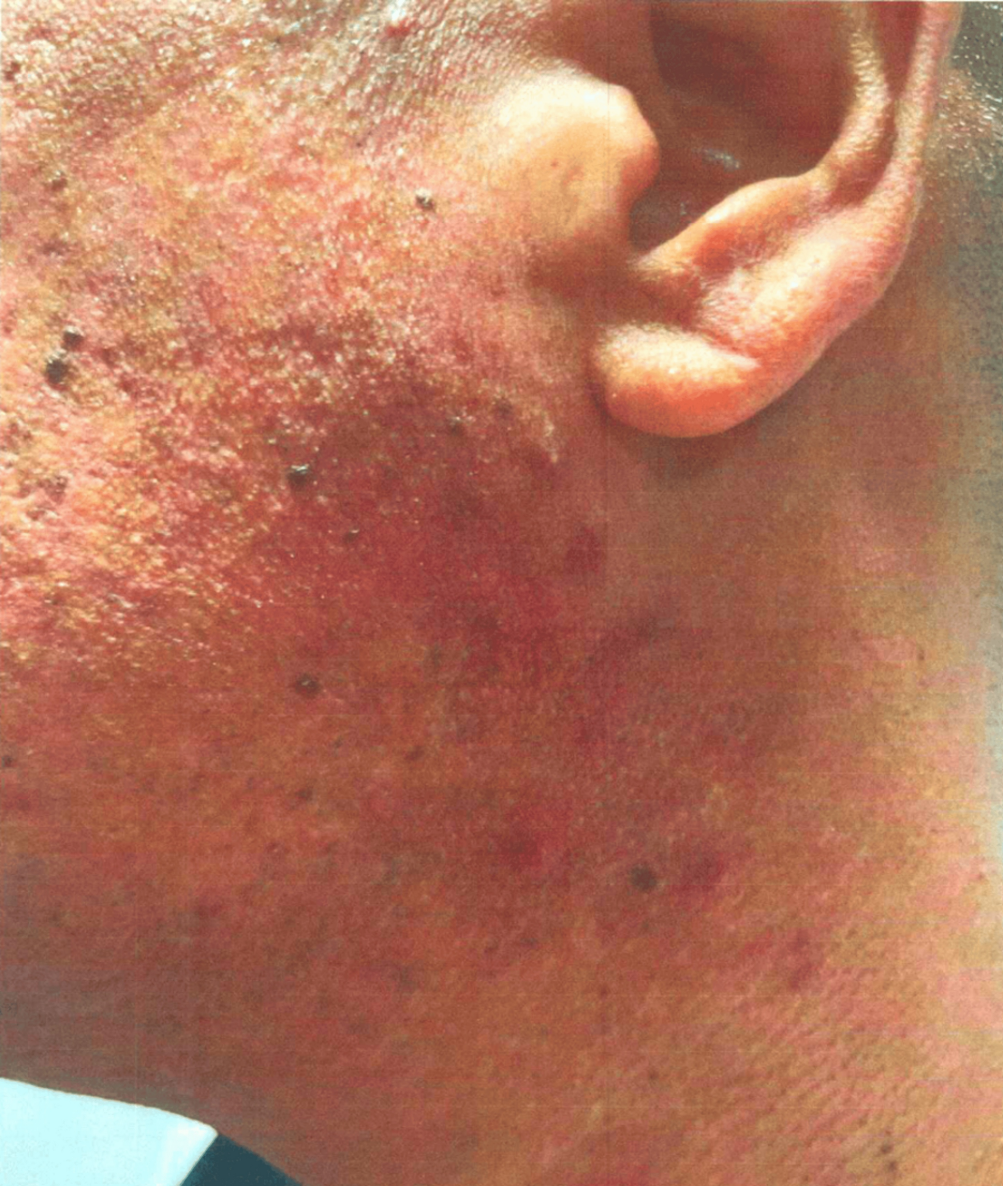
Case 2: patient's dermatologic findings in the left side of his face/neck

**Figure 4:**
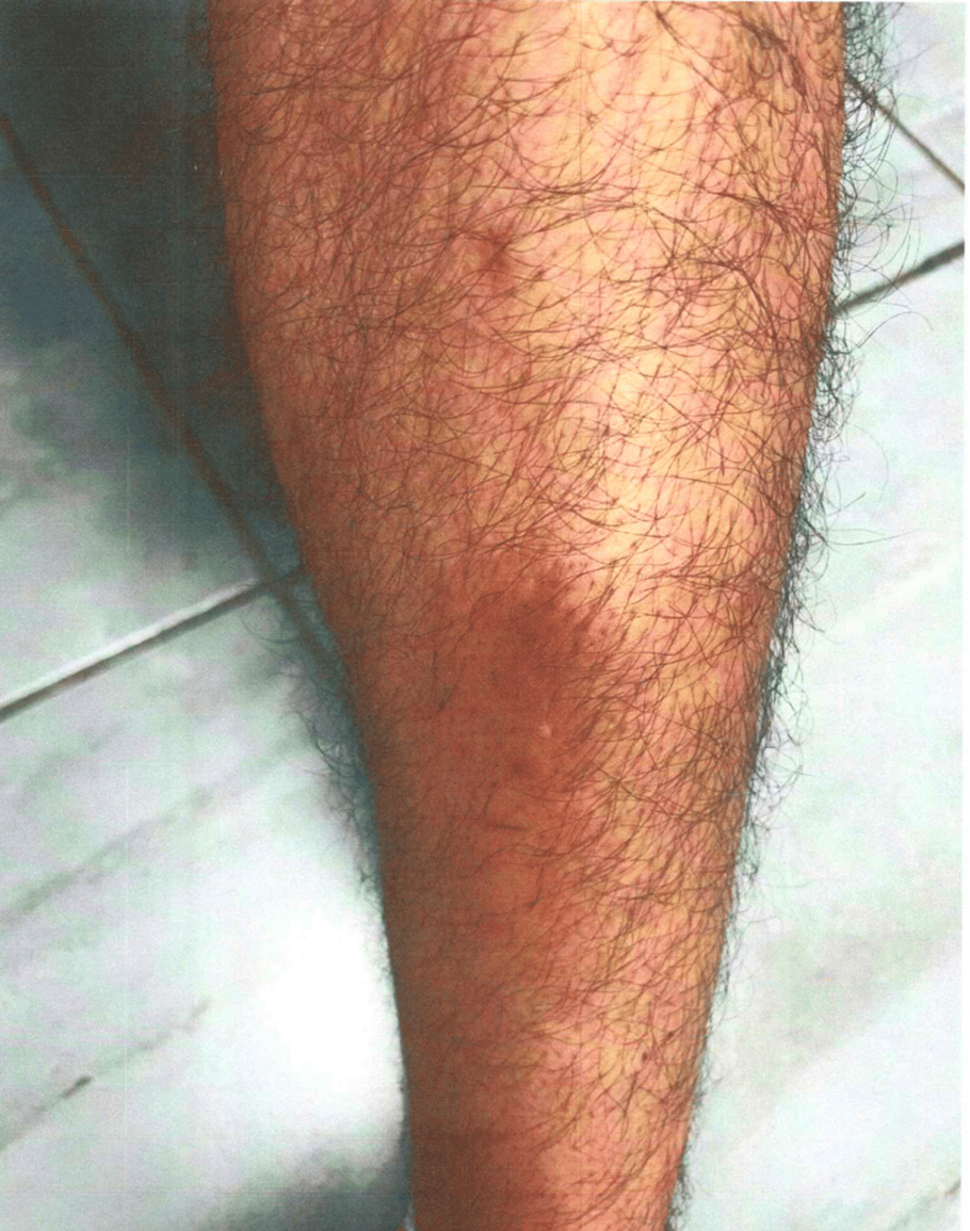
Case 2: patient's dermatologic findings in the left lower extremity

The patient's dermatologic findings are shown in Figures 3-4.

Case 3: unrelated patient with PCT and hemochromatosis

A 70-year-old male patient (unrelated to the other two cases) was diagnosed with PCT. Upon genetic testing, the patient had two pathogenic variants in the HFE gene, C282Y (c.845G>A, p.Cys282Tyr) and H63D (C.187C>G, p.His63Asp), which are commonly associated with hereditary hemochromatosis. This patient, an HFE gene mutation carrier, had symptoms of PCT, which were managed with therapeutic phlebotomy. Laboratory test results are shown in Tables 6-7.

**Table 6:** Case 3: lab results pre-phlebotomy (2010) μg: micrograms; L: liters

Laboratory test results pre-phlebotomy	
Uroporphyrins (UP)	40 μg/L (HIGH)
Heptacarboxyl (7-CP)	8 μg/L (HIGH)
Hexacarboxyl (6-CP)	<1 μg/L
Pentacarboxyl (5-CP)	<1 μg/L
Coproporphyrin (CP) I	43 μg/L (HIGH)
Coproporphyrin (CP) III	5 μg/L

**Table 7:** Case 3: lab results post-phlebotomy (2024) μg: micrograms; L: liters

Laboratory test results post-phlebotomy	
Uroporphyrins (UP)	33 μg/L (HIGH)
Heptacarboxyl (7-CP)	<1 μg/L
Hexacarboxyl (6-CP)	<1 μg/L
Pentacarboxyl (5-CP)	<1 μg/L
Coproporphyrin (CP) I	26 μg/L (HIGH)
Coproporphyrin (CP) III	52 μg/L (HIGH)

**Figure 5:**
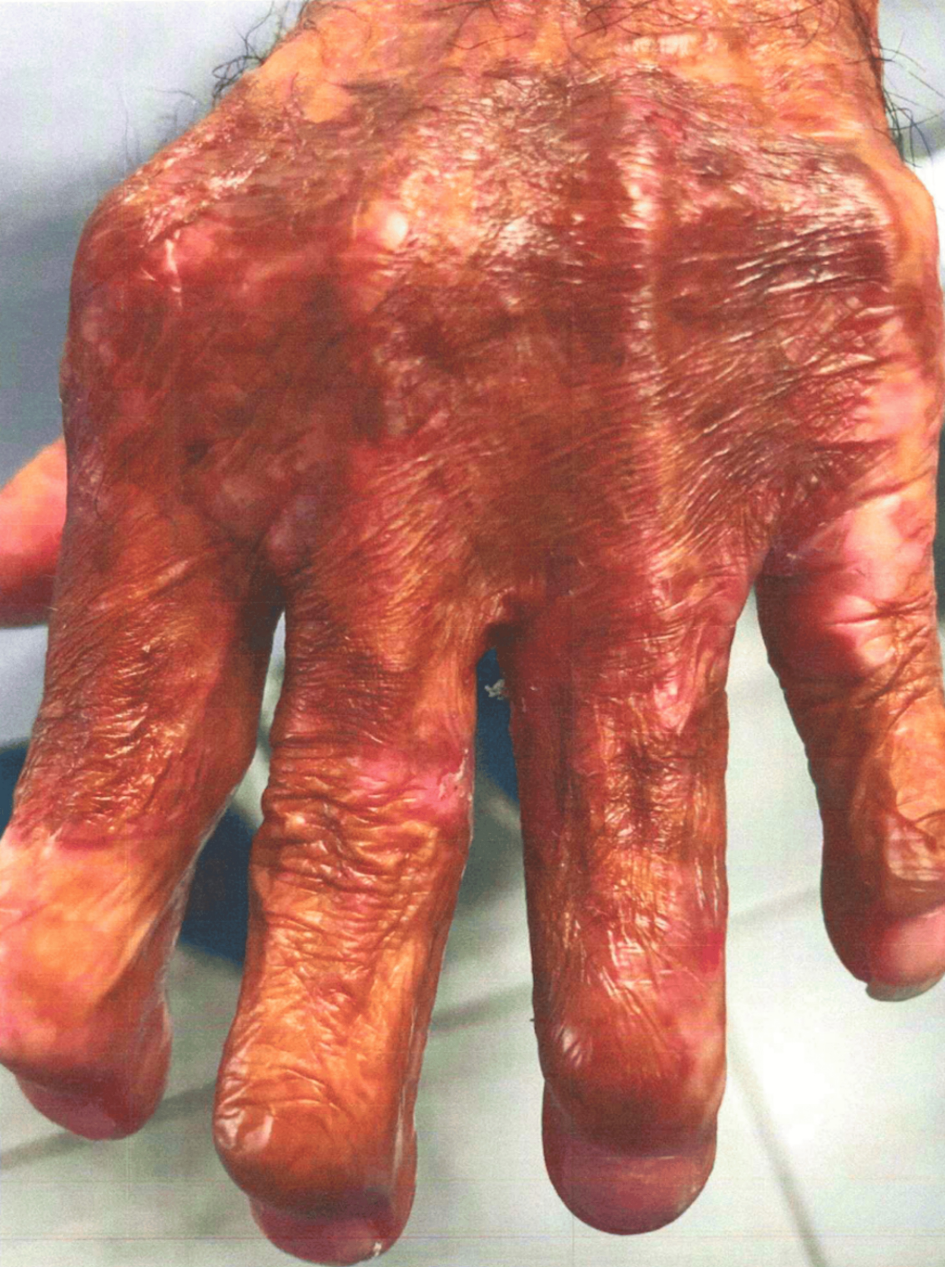
Case 3: patient's dermatologic findings on his left hand

**Figure 6:**
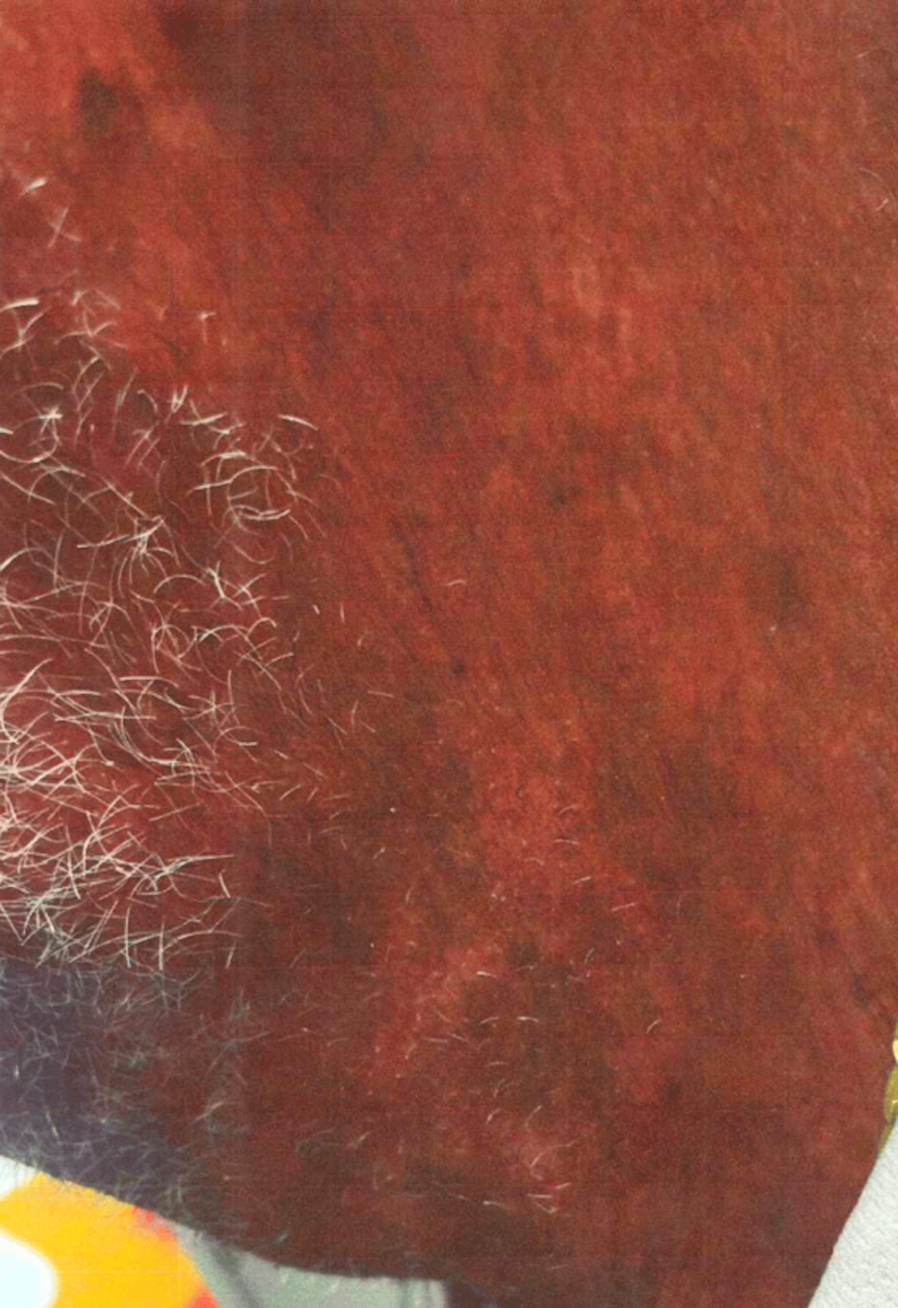
Case 3: patient's dermatologic findings in the left side of his neck

The patient's dermatologic findings are shown in Figures 5-6.

## Discussion

Porphyria is a group of diseases that are characterized by abnormalities in the heme biosynthetic pathway, leading to the accumulation of porphyrins or their precursors, which can cause a variety of symptoms, including photosensitivity, skin lesions, and neurological complications.

Several genetic mutations can lead to porphyria: EPP, PCT, acute intermittent porphyria, hereditary coproporphyria, variegate porphyria, and congenital erythropoietic porphyria [5].

EPP is most commonly caused by autosomal recessive mutations in the FECH gene, leading to the accumulation of protoporphyrins in the skin. This leads to painful photosensitivity and potential liver damage due to the biliary excretion of protoporphyrins [12]. Case 1 was clinically diagnosed with porphyria, and therapeutic phlebotomy was done to manage the patient's signs and symptoms. Upon genetic analysis, Case 1 had a heterozygous mutation in the FECH gene. This is compatible with a partial ferrochelatase activity and, thus, a less severe clinical presentation of the disease. 

Furthermore, Case 2 (son of Case 1) was previously diagnosed with PCT. Photosensitivity, a symptom of congenital erythropoietic porphyria, can also occur in the cutanea tarda and "mixed" hepatic types [13]. Genetic testing showed that the patient had no mutations in the porphyria genes. Although the patient had similar skin blistering as Case 1, the genetic findings remain inconclusive. 

Case 2 presented with solar urticaria and other acute skin changes caused by ultraviolet radiation. Additionally, the patient's uroporphyrin levels were elevated at the time of collection in 2010. These findings raise the question of whether the patient can be diagnosed with porphyria, despite a negative result on the porphyria genetic panel. To our knowledge, these findings have not been previously described. 

Hereditary hemochromatosis, often associated with PCT, is an autosomal recessive condition that leads to iron overload, further complicating the clinical management of porphyria [11,14]. Case 3 was diagnosed with PCT. The genetic assay detected two pathogenic variants in the HFE gene: C282Y and H63D. Both variants are commonly associated with hereditary hemochromatosis, which is characterized by a disorder in iron metabolism. Li and co-workers report a rare case of hereditary hemochromatosis with similar pathogenic variants in the HFE gene [15]. After regular phlebotomy, the patient's serum ferritin (SF), serum iron (SI), and transferrin saturation (TSAT) decreased. Additionally, transferrin (TF) and liver function returned to normal, and the patient's signs and symptoms improved. For our patient, therapeutic phlebotomy was performed. Post-phlebotomy, TF level was 336 mg/dL, SI 18 mg/dL, iron 30 mg/dL, iron saturation 6.9%, hemoglobin 16.9 g/dL, and hematocrit 53.6%. Case 3 of this report underscores the importance of considering genetic testing and managing iron levels in patients with PCT.

Overall, these cases illustrate the diverse presentations and complexities involved in the diagnosis and management of the porphyrias. Understanding the underlying genetic mutations and their biochemical consequences is crucial for the effective co-management, treatment, and improvement of patient outcomes.

Genetic studies in patients with porphyria are warranted. Genetic testing may provide critical insights into the diagnosis, management, and prognosis of various hematologic hereditary diseases. This is the first report of the genetics of porphyria in Puerto Rico. 

Limitations of the study include the small number of PCT cases. Further studies will continue to elucidate the genetics leading to PCT in patients, enhancing our understanding and treatment of this complex group of disorders.

## Conclusions

We report on the clinical and genetic findings of patients with porphyria. With case presentations of EPP and PCT, we underscore the complexities in diagnosing and managing these disorders, particularly in the context of genetic variability. The identification of a novel case in Puerto Rico enriches the understanding of porphyria's phenotypic expression in diverse populations. Our findings emphasize the critical role of genetic testing in elucidating the etiology of porphyria, guiding therapeutic interventions, and improving patient outcomes. Further studies should continue to explore the genetic and environmental factors contributing to porphyria, facilitating more precise diagnostic and management strategies. 
